# Template Free Synthesis of Hollow Ball-Like Nano-Fe_2_O_3_ and Its Application to the Detection of Dimethyl Methylphosphonate at Room Temperature

**DOI:** 10.3390/s120404594

**Published:** 2012-04-10

**Authors:** Guokang Fan, You Wang, Meng Hu, Zhiyuan Luo, Kaihuan Zhang, Guang Li

**Affiliations:** 1 State Key Laboratory of Industrial Control Technology, Institute of Cyber Systems and Control, Zhejiang University, Hangzhou 310027, China; E-Mails: gkfan@yzu.edu.cn (G.F.); king_wy@zju.edu.cn (Y.W.); vhumeng@126.com (M.H.); zhangkaihuan@zju.edu.cn (K.Z.); 2 School of Chemistry and Chemical Engineering, Yangzhou University, Yangzhou 225002, China; 3 Computer Learning Research Centre, Royal Holloway, University of London, Egham, Surrey TW20 0EX, UK; E-Mail: Zhiyuan.Luo@cs.rhul.ac.uk

**Keywords:** nano-Fe_2_O_3_, quartz crystal microbalance (QCM), dimethyl methylphosphonate (DMMP), gas sensor

## Abstract

This paper is focused on the template-free synthesis of nanosized ferric oxide (nano-Fe_2_O_3_) and its application in quartz crystal microbalance (QCM) resonators to detect dimethyl methylphosphonate (DMMP), a simulant of Sarin. The X-ray diffraction (XRD) patterns confirm that the synthesized samples are made of Fe_2_O_3_ and the scanning electron microscopy (SEM) pictures show that the samples have ball-like shapes. The DMMP sensors with a sensing film of hollow ball-like and solid ball-like Fe_2_O_3_ are fabricated and their sensing characteristics are compared. The sensitivity of the hollow ball-like Fe_2_O_3_ sensor is more than 500% higher than the one of the solid ball-like Fe_2_O_3_ sensor. The hollow ball-like nano-Fe_2_O_3_ can be synthesized by a novel low temperature hydrothermal method. The sensors with the hollow ball-like Fe_2_O_3_ film perform well in a range of 1 to 6 ppm, with a sensitivity of 29 Hz/ppm at room temperature, while the appropriate recoverability and selectivity are maintained. In addition, the performance of different thicknesses of the sensing film of the hollow ball-like nano-Fe_2_O_3_ is investigated and the optimized relative film thickness of the hollow ball-like nano-Fe_2_O_3_ is found to be 20 μg/mm^2^.

## 1. Introduction

Recently, fast and easy detection of dimethyl methylphosphonate (DMMP) has been the focus of much research [1–6]. Due to its similar molecule structure to Sarin, a kind of dangerous chemical warfare agent, DMMP is commonly considered as a simulant of Sarin [7–10]. Several methods have been developed to detect DMMP at the ppm level [4,7,8,11–13]. Because of their high sensitivity and rapid response as well as their room temperature working conditions, quartz crystal microbalance (QCM)-based sensors have become the most popular choice [14–18]. For a QCM sensor, the selectivity is determined by the sensing film material. Therefore, the key challenge for a QCM gas sensor is how to adopt an appropriate sensing film material for the analyte to be detected.

In order to improve the selectivity of QCM sensors, various sensing materials have been investigated [7,9,14–18]. In particular, nano-structured and hybrid materials [16] are studied for use as sensing materials because they have large specific surface areas due to their ultra-fine grain.

In this paper, a kind of hollow ball-like nano-Fe_2_O_3_ is synthesized via a simple template-free method and used as the sensing material to detect DMMP. The thickness of the sensing film is optimized. The sensitivity and selectivity of the sensor with the optimized sensing film thickness are also investigated.

## 2. Experimental Section

### 2.1. Materials

DMMP (dimethyl methylphosphonate) was purchased from Qindao Hanhua Fireproofing Material Ltd., China. Ferric trichloride (FeCl_3_·6H_2_O), ammonia solution, anhydrous methanol, anhydrous alcohol trichloromethane and *n*-hexane were all AR grade. The HC-49/U AT-cut 6.0 MHz quartz crystals were from Hosonic International (Hangzhou) Ltd., China.

### 2.2. Methods

#### 2.2.1. Preparation of Hollow Ball-like and Solid Ball-like Nano-Fe_2_O_3_

In a typical procedure, 0.1 mol·L^−1^ FeCl_3_ solution (30 mL) prepared from FeCl_3_·6H_2_O and deionized water was placed in a 100 mL glass vessel. Diluted aqueous ammonia solution (2 mL, 1:1 v/v) was gradually injected into the continuously stirred solution and brown milk-like ferric hydroxide precipitate was obtained. This was then stirred for 30 minutes and aged for 24 hours at room temperature.

- Hollow ball-like nano-Fe_2_O_3_ synthesis: The brown milk-like ferric hydroxide precipitate was put into a 100 mL autoclave and treated in a 90 °C oven for 3 hours. After cooling down to room temperature, the upper liquid layer was recycled. The deposit was washed with deionized water five times and dried at a 100 °C oven to obtain a reddish brown powder.
- Ball-like Fe_2_O_3_ synthesis: after the sample was aged again for 24 hours (total 48 hours), the clear upper liquid was recycled. After replacing with deionized water, the mixture was moved to a 100 mL glass vessel and treated in a 90 °C oven for 3 hours. The deposit was washed with deionized water five times and dried in an 80 °C oven to obtain a brown powder.

#### 2.2.2. Characterization of the Fe_2_O_3_ Samples

Scanning electron microscopy (SEM) observation was performed with an S4800 system (Hitachi, Tokyo, Japan). The Fe_2_O_3_ samples were coated on the Ag electrodes of AT-cut 6.0 MHz quartz crystals. The X-ray diffraction (XRD) patterns were recorded on an X-ray diffraction device (X'Pert PW3050/60, PANalytical, Sugapore) running with Cu Kα radiation in an angle degree range from 20° to 80° (2θ).

#### 2.2.3. Fabrication of a QCM Gas Sensor

After an AT-cut 6.0 MHz quartz crystal was unshelled, typically, 4 μL of sample mixture (the concentration was 100 ± 5 mg·mL^−1^) was dispensed onto a Ag electrode of one side. Then the quartz crystal was dried at room temperature for 24 hours.

#### 2.2.4. Gas Sensing Experiment

The gas sensing response was measured in a 500 mL sealed chamber. One coated quartz crystal was the sensing QCM and the other non-coated one was the reference one [19,20]. The frequency difference between them was recorded every second by a personal computer (PC) via a RS-232 serial communication port. When a gas sample, such as DMMP, was injected into the chamber, the change of frequency difference was recorded as the sensor response. Before each test, a new QCM sensor should be purged in the chamber with alternating high-purity N_2_ and N_2_ diluted sample gas at least five times. After every test, the sensor had to be purged with high-purity N_2_. The detailed experimental set-up can be found in our previous work [20].

## 3. Results and Discussion

### 3.1. Template Free Synthesis and the Detection at Room Temperature

Recently, Buathong and co-workers reported a template-free synthesis of nano-Fe_2_O_3_ particles with diameters of about 39 nm, but the synthesis has to be carried out at 250–300 °C under argon protection [21]. Rangaraju and co-workers synthesized iron oxide nanotubes on a pure iron substrate by an electrochemical anodization method. The grain size is in the range of 80–110 nm. When sodium tetraborate and sodium tetrafluoroborate were used, annealing at 500–550 °C was indispensable [22]. The synthesis including a 200–450 °C heating treatment process was reported in the references [23,24] as well. Instead of room temperature, the best detection temperature was 230 °C, as described in the reference [25].

For the experiments reported in this paper, the highest temperature for synthesis was as low as 100 °C, and all detection measurement were carried out at room temperature, so both the synthesis and measurement temperatures were much lower than those reported in the references [23,24]. Furthermore, several environmentally friendly concepts were introduced throughout our experiments such as the relatively low temperature reaction, the recycle of the clear upper liquid and the detection at room temperature. The synthesis quality could be improved by further study and the synthesis conditions could also be optimized to obtain uniform grains.

### 3.2. XRD Diffraction

**Figure 1.**
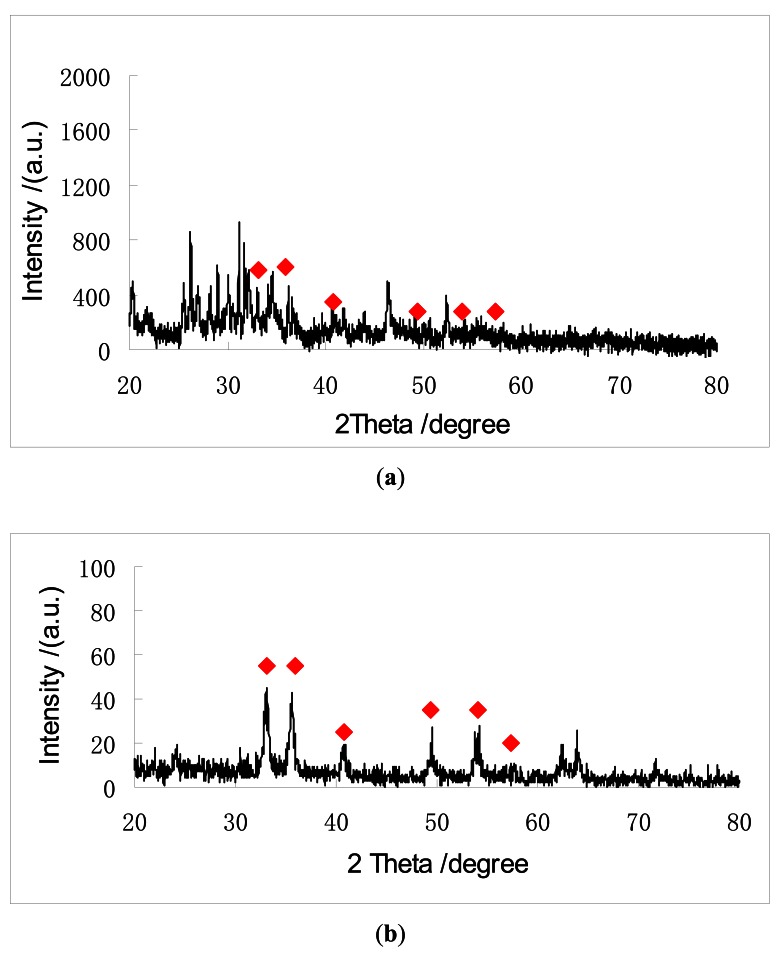
The X-ray diffraction pattern of the Fe_2_O_3_ samples: (**a**) Hollow ball; (**b**) Solid ball.

The XRD patterns of the hollow and solid ball-like Fe_2_O_3_ samples are shown in Figures 1(a) and 1(b), respectively.

The diffraction peaks in 33.1, 36, 40.8, 49.4, 54 and 57.4 degrees as labeled in Figure 1(a,b) are well matched with the characteristic peaks of α-Fe_2_O_3_ [24], so the spectra indicate the samples are mostly made of Fe_2_O_3_. Due to the drying at room temperature in air, the samples suffered from poor crystallinity and humidity. As a result, background peaks can be seen in Figure 1. But this will not affect the sensing tests, because the specific surface area and morphology of the material are the most important factors. Better XRD patterns could be obtained by annealing [24,25], but the QCM would suffer from such high temperatures. In order to save energy and simplify the processes, the same experimental conditions were used in both XRD and QCM gas sensing experiments.

### 3.3. SEM Morphology

**Figure 2.**
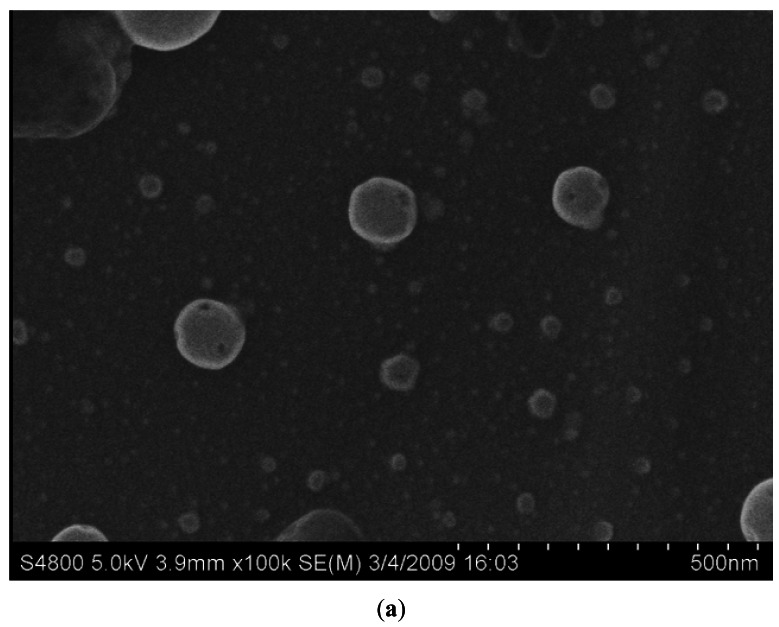

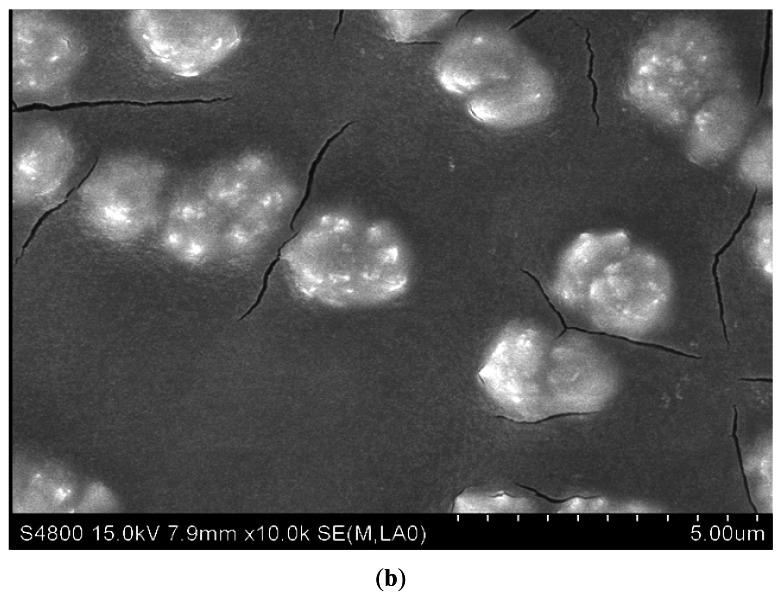
The SEM images of the Fe_2_O_3_ samples: (**a**) Hollow ball; (**b**) Solid ball.

In Figure 2(a,b), Fe_2_O_3_ samples exhibit a hollow and solid ball-like morphology, respectively. The hollow balls have a diameter range from 20 nm to 120 nm, and the solid balls are of 200–800 nm. Figure 2(a,b) also illustrates that most of hollow ball samples are of nanometer scale, but their solid ball counterparts are not. The solid balls are just used for the comparison. The larger the diameter, the smaller is the specific surface area, so the wide range of the solid ball diameters will not affect the comparison results. Because the hollow and solid balls have the same total mass on sensors' films, it is obvious that the hollow balls have larger specific surface area when they have the same diameter. Moreover, for ball-like Fe_2_O_3_, the larger diameters mean less specific surface area for the same mass, so the specific surface area of the hollow ball-like nano-Fe_2_O_3_ film is larger than the solid ball-like one when the hollow ball's diameter is less than that of the solid ones.

### 3.4. Repeatable Response to DMMP

**Figure 3.**
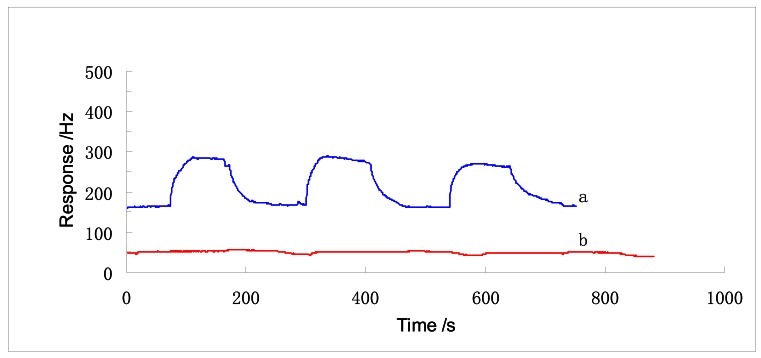
The reversible response curve to 4 ppm DMMP: (**a**) Hollow ball; (**b**) Solid ball.

For the sake of simplicity, the sensors with the sensing films of hollow ball-like nano-Fe_2_O_3_ and solid ball-like Fe_2_O_3_ are called SENSOR No.1 and SENSOR No.2, respectively. SENSOR No.1 has a rapid (25 s) and high (115 Hz) response to 4 ppm DMMP, as shown in Figure 3.

According to the report of Tasaltin and coworkers, their QCM sensor's response time to 12 ppm DMMP was 18 minutes and response amplitude to 6 ppm DMMP was lower than 100 Hz [7]. Also the response time of our SENSOR No.2 is about 20 s, but the amplitude is a little bit lower than 20 Hz. As a result, SENSOR No.1 has a sensitivity of 29 Hz/ppm, which is at least 500% more sensitive than SENSOR No.2. Ying and coworkers reported that their DMMP sensor's sensitivity was 3.19 Hz/ppm [9]. Since the responses of the two sensors, SENSOR No.1 and SENSOR No.2, are repeatable, N_2_ is a successful desorption gas. Because adsorption of N_2_ is physical, namely, unspecific surface absorption, the successful desorption of DMMP suggests that the DMMP adsorption in the sensing film is not chemical, or it would not be desorbed well by N_2_, so the intermolecular force between the gas and the sensing material must be the main interaction force, which can explain why the SENSOR No.1 can have higher response amplitude. Because of the larger specific surface area, SENSOR No.1 has more surface energy than SENSOR No.2.

### 3.5. Responses of the SENSOR No.1 to Different DMMP Concentrations

**Figure 4.**
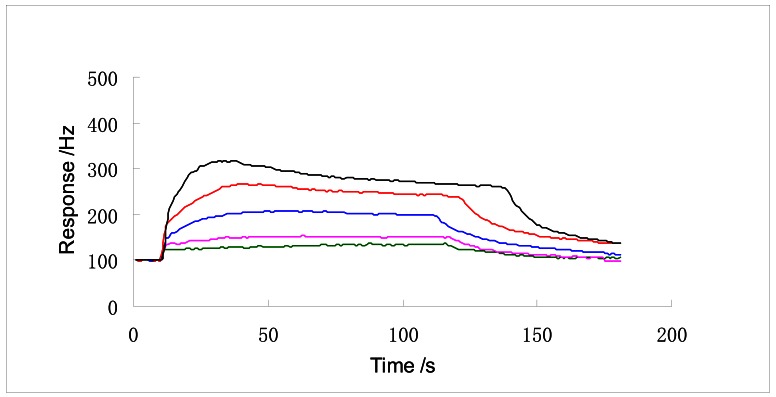
The response curves to 1, 2, 4, 6 and 10 ppm DMMP (from bottom to top).

Figure 4 shows the responses of SENSOR No.1 to DMMP with concentrations from 1 to 10 ppm, which illustrates that the responses are gradually enhanced with the increasing concentrations of DMMP. It is important to note that there is a linear response from 1 to 6 ppm.

### 3.6. The Thickness of the Sensing Film and Response Properties

**Figure 5.**
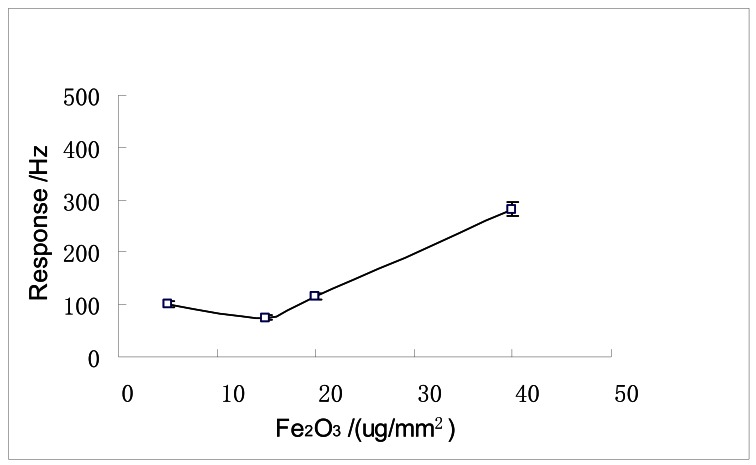
The response curve of various sensing film thicknesses of SENSOR No.1 to 4 ppm DMMP.

In Figure 5, the smallest response is observed when the relative film thickness (μg/mm^2^) of the hollow ball-like nano-Fe_2_O_3_ is 15 μg/mm^2^. This is different from the result in reference [19], which reported that the trend of response amplitude to relative film thickness is firstly increasing to a maximum and then dropping. Our test results in this paper are due to the hollow property of the nano-Fe_2_O_3_. The 15 μg/mm^2^ film can only cause monolayer saturated gas adsorption. Meanwhile, the 5 μg/mm^2^ film leads to a direct multilayer adsorption. The 20 μg/mm^2^ film is initially monolayer saturated adsorption and then multilayer one. Because the 40 μg/mm^2^ film has the largest mass comparing to the others, with the similar adsorption feature to 20 μg/mm^2^ film, it has the highest response amplitude to DMMP. Nevertheless, the 40 μg/mm^2^ sensor can cause a long recovery time of the sensor, which has been observed in our experiments. Considering both the response amplitude and the recovery time, 20 μg/mm^2^ film (a balanced condition) was adopted as the optimized thickness based on our experimental results.

### 3.7. Selectivity of the SENSOR No. 1

**Figure 6.**
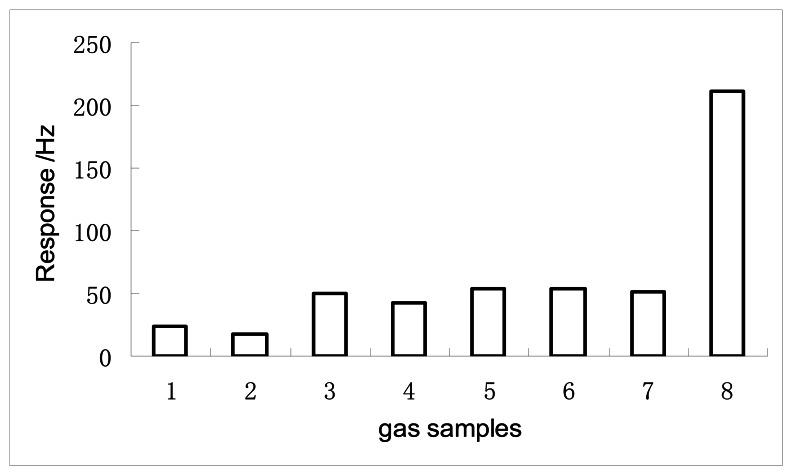
The response of the sensor to different 10 ppm sample gases (from left to right). 1, methanol; 2, ethanol; 3, formal dehyde; 4, acetal dehyde; 5, ethyl acetate; 6, n-hexane; 7, chloroform; 8, DMMP.

Figure 6 depicts the response amplitude of the SENSOR No.1 to eight different kinds of gases with 10 ppm concentration.

In the graph bar 1 is methanol, 2 is ethanol, 3 is formaldehyde, 4 is acetaldehyde, 5 is ethyl acetate, 6 is *n*-hexane, 7 is chloroform and 8 is DMMP. The response amplitude of DMMP is much higher than those of other gases, which indicates that the sensor has an excellent selectivity towards DMMP. Since the P=O function group may easily interact with the -OH groups of the nano-Fe_2_O_3_ surface by hydrogen bond [12,15], the response of the sensor to DMMP is better than the others.

## 4. Conclusions

Hollow ball-like nano-Fe_2_O_3_ was synthesized by a template-free hydrothermal method at a low temperature. After the relative thickness of the sensing film was optimized to 20 μg/mm^2^, the DMMP sensors based on the Fe_2_O_3_ sensing films were investigated. As results, the sensor with the hollow ball-like nano-Fe_2_O_3_ has a sensitivity of 29 Hz/ppm to DMMP, while the appropriate recoverability and selectivity were maintained. Comparison tests indicated that the hollow ball-like nano-Fe_2_O_3_ is more sensitive than the solid ball-like one. Our experimental results showed the sensitivity of the hollow ball-like material is more than 500% higher than that of the solid ball-like one. Therefore, the hollow ball-like nano-Fe_2_O_3_ is a promising sensing material to fabricate Sarin sensors which work at room temperature.
